# Assessment of Physicians’ Practice in Implementing Antibiotic Stewardship Program in Najran City, Saudi Arabia: A Cross-Sectional Study

**DOI:** 10.3390/pharmacy12010024

**Published:** 2024-02-01

**Authors:** Nasser Saeed Alqahtani, Maha Mohammed Bilal, Albatoul M. Al Margan, Fatimah Ahmad Albaghrah, Anwar Mana Al Sharyan, Aljawharh Salem M. Alyami

**Affiliations:** 1 Department of Family and Community Medicine, College of Medicine, Najran University, Najran 66462, Saudi Arabia; 2 Medical Intern, College of Medicine, Najran University, Najran 66462, Saudi Arabia

**Keywords:** antimicrobial stewardship program, practice, effectiveness, primary physicians, Saudi Arabia

## Abstract

Introduction: One of the main causes of illness, mortality, and rising medical costs is antimicrobial resistance, which is a global healthcare concern. Objectives: This study explores the practice of physicians toward the effective implementation of Antibiotic Stewardship Programs (ASPs) in Najran city, Saudi Arabia. Methodology: This cross-sectional study was conducted among physicians working at primary care setting in Najran city, Saudi Arabia, between May and August 2023. A self-administered questionnaire was distributed among the physicians composed of three parts: socio-demographic data, a questionnaire about physicians’ practice in the efficacy of ASP, and a questionnaire about physicians’ practice regarding prescribing antibiotics. Results: Of the 128 physicians who participated in the study, 60.2% were males, and 43.8% were aged between 36 and 45 years. Among the practices in implementing the ASP effectively, controlling the source of infection domain received the highest score (mean score: 4.83). Every practice domain mean score was greater than 3, indicating that study participants possessed a moderate level of ASP practice and implementation skills. The overall mean practice score in the effective implementation of ASP was 154.9 ± 25.5 out of 185 points, with good, moderate, and poor practices constituting 67.2%, 28.1%, and 4.7%, respectively. Conclusions: The physicians showed a moderate level of practice for the effective implementation of ASPs in Najran city. The factors significantly associated with increased practice score include older age, male gender, Saudi nationality, handling five or fewer infection cases daily, and infection-initiated antibiotic prescribing treatment managed per day. These findings suggest the need for targeted interventions and educational programs to enhance physicians’ adherence to ASP guidelines and promote appropriate antibiotic prescribing practices, ultimately contributing to global efforts in combating antimicrobial resistance and improving patient outcomes.

## 1. Introduction

One of the main causes of illness, mortality, and rising medical costs is antimicrobial resistance, which is a global concern in healthcare. The excessive and inappropriate use of antibiotics in hospitals and in the community is the main cause of resistance [1]. Between the year 2000 and 2015, the global use of antibiotics climbed by 65%, with most of this growth occurring in low- and middle-income countries [2]. The WHO released the global action plan in 2015 to overcome the threatening concerns of antibacterial resistance. An integral part of this plan is the Antibiotic Stewardship Program (ASP), a program that ensures that antibiotics are used appropriately, both in humans and in animals [1,3]. The Antibiotic Stewardship Program aims to enhance infection cure rates, minimize toxicity and adverse events, prevent antibiotic resistance, and improve the quality of patient care while also reducing healthcare costs [4,5].

The role of physicians in promoting ASP is crucial. By adhering to guidelines, staying informed, collaborating with the healthcare team, and educating patients, physicians can contribute to reducing antimicrobial resistance and improving patient outcomes. As the frontline providers of medical care, physicians play a crucial role in promoting responsible antibiotic use and ensuring the effectiveness of these life-saving medications.

An international cross-sectional survey of antimicrobial stewardship programs in 660 hospitals across 67 countries conducted by Howard et al., 2015, reported that insufficient funds and personnel, as well as physician disagreement, were the main barriers to ASPs in hospitals [6]. It was noted that many doctors had misconceptions about the rational use of antibiotics, and Antibiotic Stewardship Programs were perceived poorly [3]. A study conducted in two university hospitals in Egypt found that most physicians and clinical pharmacists had limited awareness of ASPs and inconsistent knowledge regarding their usefulness, efficacy, and applications in ASPs [7]. In Bangladesh, a recent study has found the irrational use of antimicrobials to be prevalent among many physicians, which showed that 81% of the prescriptions had at least two antibiotics [8]. Furthermore, physicians often express concerns about the implementation of ASPs as they fear it may result in a loss of their autonomy in prescribing antimicrobial medications. ASPs often establish a restricted formulary, which limits the available antibiotic options for physicians. They may need to adhere to specific guidelines and protocols when selecting antibiotics, restricting their freedom to prescribe broader-spectrum or newer antibiotics [9].

In Saudi Arabia, hospitals and healthcare facilities are experiencing a growing problem with antimicrobial resistance and the emergence of multi-drug resistant strains [10]. This poses a significant threat to the effectiveness of treatments and patient outcomes. The misuse of antimicrobials is contributing to this crisis, highlighting the urgent need for interventions to preserve antimicrobial effectiveness and reduce resistance rates [10]. ASPs have proven to be effective in improving antimicrobial usage, enhancing treatment success rates, and mitigating the development of antimicrobial resistance [11]. To address these challenges, the Saudi Ministry of Health (MOH) has developed a national antimicrobial stewardship plan aimed at implementing ASPs in hospitals.

A recent study in the Eastern province of Saudi Arabia on ASP revealed that more than 50% of clinicians reported a lack of awareness of ASP, whereas 71.2% do not have previous ASP experience [12]. According to another qualitative study conducted in Saudi hospitals, barriers to the adoption and implementation of ASPs include concerns among physicians regarding their liability, as well as a lack of enforcement of policies and guidelines by governing bodies [13].

Multiple global surveys have reported the successful implementation of various antimicrobial stewardship measures in healthcare institutions in Saudi Arabia. However, these studies have not specifically addressed the clinical practice of physicians regarding the adoption of ASPs in Saudi Arabia. In addition, this study is one of the first to be conducted in Southern Saudi Arabia that evaluates physicians’ participation in implementing ASPs. Hence, building upon previous research, this study aims to explore the practice of physicians towards effective implementation of ASPs at primary care settings in Najran city, Saudi Arabia. The primary objectives of the study are as follows: (1) to evaluate the level of physician engagement in the effective implementation of ASPs; (2) to identify key predictors that influence participation in the effective implementation of ASPs; (3) to examine physicians’ practices regarding the prescription of antibiotics in Najran city. By investigating these aspects, the study seeks to contribute to the understanding of physicians’ practices in relation to ASPs in Saudi Arabia and identify factors that may influence their engagement in ASP implementation.

In summary, the rationale for this study lies in the significance of addressing antimicrobial resistance concerns, understanding and improving physicians’ practices related to ASPs, and providing region-specific insights to enhance ASP implementation in Najran city, Saudi Arabia. The study aims to contribute to the body of knowledge on ASPs and guide future interventions to improve patient care and combat antimicrobial resistance. The research aligns with global efforts to combat antimicrobial resistance and improve the effectiveness of antibiotic treatments through comprehensive ASPs.

## 2. Methodology

### 2.1. Study Design and Population

For the assessment of physicians’ participation in the effective implementation of ASPs, an observational cross-sectional design was used. The study was conducted from May to August 2023 in Najran city, which is located in the southern region of Saudi Arabia. There are more than 50 healthcare facilities, including 32 primary healthcare centers, 10 tertiary hospitals, and many private polyclinics.

### 2.2. Sample Size, Sampling Method, and Data Collection

The total number of physicians working in different hospitals in Najran city is 400 approximately. The final minimum sample size of 129 participants was determined using the Raosoft sample size calculator (http://www.raosoft.com/samplesize.html (accessed on 15 April 2023)). The calculation was based on the formula x = Z(c/100)2r(100 − r), where N represents the population size, r represents the fraction of responses, and Z(c/100) represents the critical value for the desired confidence level (c). To calculate the required sample size, we assumed a precision level of 5% and a margin of error of 6%. Additionally, a confidence level of 95% was chosen to ensure the desired level of statistical significance. By using simple random sampling, we recruited physicians working at primary care settings in health facilities in Najran city. A total of 200 physicians from 20 health facilities were approached, of whom 128 responded positively and gave consent to participate in the study, with a response rate of 64%.

### 2.3. Construction, Validation, and Reliability of Study Tool

In order to design the first version of the questionnaire, an extensive review of the relevant literature [1,3,4,5,6,7,8,9,12,13] was carried out. Subsequently, experts in the fields of community medicine, pharmacy, and epidemiology were consulted to ensure the content validity of the questionnaire. The input and feedback from these experts were taken into consideration during the comprehensive revision process. We designed a questionnaire that consisted of 9 domains covering the duties of physicians to successfully implement the ASP as reported by The Global Alliance for Infections in Surgery [14]. To establish face validity, a pilot study involving 20 participants was conducted. The responses obtained from the pilot study were included in the final data analysis. Moreover, the reliability of the questionnaire was assessed, and Cronbach’s alpha coefficient was calculated for the questionnaire with 37 items. The obtained Cronbach’s alpha value of 0.8 indicated satisfactory reliability of the questionnaire.

The questionnaire was self-administered and consisted of 3 parts. The first part contained the demographic data which includes the physician’s age, gender, family status, nationality, job title, position, qualifications, healthcare facility, workplace, and experience. The second part was about the physician’s participation in the efficacy of ASP implementation, which included 37 tasks divided into 9 responsibilities. Participants were encouraged to self-report his/her practice using the Likert scale (always, often, sometimes, rarely, and never). The third part included the practice of physicians regarding the prescribing of antibiotics, including the number of infection cases, the number of infection cases managed with symptomatic treatment (no antibiotic), the number of infection cases managed with delayed antibiotic prescribing, and the number of infection cases managed with initiated antibiotic prescribing. Participants labeled his/her responses for this part with the average number managed per day.

### 2.4. Ethical Clearance

The ethical approval was taken from the Najran University Research Ethics Committee (CSR/NU/2022/1022). All fundamental ethics were maintained for participants, including autonomy, confidentiality, data security, and justice. The study’s objectives, along with the written informed consent form, were presented at the start of the questionnaire. Once individuals had read the consent document and understood the purpose of the study, they had the freedom to decide whether to proceed with participation or decline. Participation was entirely voluntary. Participants were provided with information about the confidentiality of their identity, ensuring that their personal information would be kept private. It was also made clear that the collected data would be used solely for research purposes.

### 2.5. Questionnaire Criteria

The practice of physicians toward ASP has been assessed using a 37-item questionnaire divided into 9 domains, namely enhancing infection prevention control, controlling source of infection, prescribing antibiotics when they are truly needed, prescribing appropriate antibiotics with adequate dosages, reassessing antimicrobial treatment when culture results are available, using the shortest duration of antibiotics based on evidence, educating staff, supporting surveillance of antimicrobial resistance (AMR) and healthcare-associated infections (HAIs), monitoring antibiotic consumption, and supporting an interdisciplinary approach, with 5-point Likert scale categories ranging from “Never” coded with 1 to “Always” coded with 5 as answer options. Statements for each domain were summed up and divided by the number of statements to obtain the mean score. The total practice score has been calculated by adding all 37 items. A possible score ranging from 37 to 185 points has been generated. As the practice score increased, the level of effectiveness in implementing ASPs also increased. A total of 60% and 80% were used as the cutoff points to determine the level of practice. Physicians were considered as having poor practice if the score was below 60%, 60% to 80% were considered moderate, and above 80% were considered good practice levels.

### 2.6. Statistical Analysis

All categorical data are shown as frequencies and proportions (%). All continuous data were calculated and summarized as mean and standard deviations. The practice score was compared with the socio-demographic characteristics and practice of prescribing antibiotics using the Whitney Z-test and the Kruskal–Wallis H-test. The normality test (i.e., statistical collinearity) was performed using the Shapiro–Wilk test as well as the Kolmogorov–Smirnov test. The total practice score follows the non-normal distribution. Therefore, the non-parametric tests were applied. Statistical significance was established at *p* < 0.05 level. All statistical data were performed and analyzed using SPSS version 26 (Statistical Packages for Social Sciences, Armonk, NY, USA: IBM Corporation).

## 3. Results

The main aims of this study were to assess the level of physician engagement in the effective implementation of Antimicrobial Stewardship Programs (ASPs) in Najran city, evaluate the key factors that contribute to physician participation in the efficacy of ASP implementation, and examine the prescribing practices of physicians concerning antibiotics in Najran city.

A total of 128 physicians participated in this study. Among them, 43.8% were in the age range of 36 to 45 years. The majority of participants were male, accounting for 60.2% of the sample. A significant proportion of the physicians were married, comprising 72.7% of the participants. Furthermore, the study included a notable number of non-Saudi physicians, making up 68.9% of the sample. Approximately 69.4% were medical practitioners, and 33.6% were diploma holders. Physicians who were working in hospital outpatient clinics constituted 44.3%. The most common workplace was MOH (54%). In addition, 31.3% had 1 to 5 years of working experience (Table 1).

Further, we assessed the physicians’ practice in the efficacy of ASP consisting of 9 domains (Table 2). Regarding enhancing the infection prevention control domain, the rating was higher for the statement, “I prevent healthcare-associated infections from occurring in the first place”. The rating for controlling the source of infection domain was higher for the statement, “I look for the septic source as early as possible” (mean score: 4.86). For the domain of prescribing antibiotics when they are truly needed, the rating was highest for the statement, “I use antibiotics when there is a high degree of suspicion of infection” (mean score: 4.59). For prescribing appropriate antibiotics with adequate dosages domain, the rating was highest for the statement, “I take into account the previous antibiotic when initiating empirical antimicrobial therapy” (mean score: 4.57). For the domain of reassessing antimicrobial treatment when culture results are available, the statement “I reassess antibiotic therapy when the culture and susceptibility results are available” (mean score 4.69) showed the highest rating. For the domain of using the shortest duration of antibiotics based on evidence, the statement “For patients who have signs of sepsis beyond 5 days of treatment, I consider warranting aggressive diagnostic investigation to determine if an ongoing uncontrolled source of infection or antibiotic treatment failure is present” (mean score: 4.55) showed the highest rating.

For the educating staff domain, the mean score for receiving training/orientation in ASP was 3.54; and for receiving feedback to improve prescribing behavior, the mean score was 3.66, indicating a moderate level of agreement. However, nearly a quarter of the participants said they “never” or “rarely” received training/orientation in ASP (24.2%) and some participants received feedback to improve prescribing behavior (15.6%). Similarly, the mean score for having training that equips the required confidence, skills, and expertise in antibiotic management was 3.81. While the mean score suggests a moderate level of agreement, a substantial number of respondents disagreed (13.3%).

Regarding the surveillance and monitoring of AMR and HAIs domain, the mean scores for supporting surveillance of antimicrobial resistance (AMR) control, healthcare-associated infections (HAIs), and notifying the Antibiotic Stewardship Program of any AMR and HAIs detected were 3.48, 3.47, and 3.56, respectively. The majority of respondents said they “often” or “always” were involved in supporting the surveillance and monitoring of AMR and HAIs; however, a considerable proportion (around one-third) of the respondents said they “never” or “rarely” supported the surveillance and monitoring.

**Figure 1:**
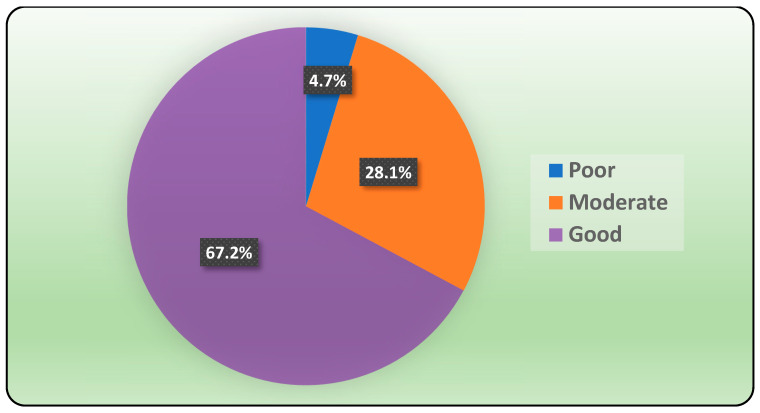
Level of physicians’ practice in the effective implementation of ASPs in Najran city.

Finally, for the domain of supporting an interdisciplinary approach, the statement “Infection control department/unit monitor and prevent healthcare-associated infections,” showed the highest mean score (mean score: 4.27). Based on the above statements, the overall mean practice score was 154 ± 25.5, with good, moderate, and poor practices constituting 67.2%, 28.1%, and 4.7%, respectively (Figure 1).

The results of the physicians’ practices regarding prescribing antibiotics are presented in Table 3. In terms of the average number of infection cases managed daily, 40.6% of physicians reported handling up to five cases. Regarding the average number of infection cases managed daily with symptomatic treatment (no antibiotic), the majority of physicians (64.1%) reported managing up to five cases. For the average number of infection cases managed daily with delayed antibiotic prescribing treatment, a significant proportion of physicians (80.5%) reported managing up to five cases. Regarding the average number of infection cases managed daily with initiated antibiotic prescribing treatment, 62.5% of physicians reported managing up to five cases.

These findings provide an overview of the physicians’ practices in terms of the number of infection cases managed daily and the approach taken regarding antibiotic prescribing, including symptomatic treatment, delayed antibiotic prescribing, and initiated antibiotic prescribing treatment.

The mean number of infection cases managed daily by physicians was 13.5, indicating that, on average, physicians deal with a moderate number of infection cases each day. The average number of infection cases managed daily with symptomatic treatment (no antibiotic) was 5.98, suggesting that physicians commonly opt for symptomatic treatment alone for a significant number of infection cases, indicating a conservative approach to antibiotic prescribing. The average number of infection cases managed daily with delayed antibiotic prescribing treatment is 3.58. This finding indicates that physicians often employ a strategy of delayed antibiotic prescribing, where antibiotics are prescribed but their use is deferred unless symptoms worsen or fail to improve within a specified period. The average number of infection cases managed daily with the initiation of antibiotic prescribing treatment is 6.43. This suggests that physicians frequently initiate antibiotic treatment for a number of infection cases. While antibiotic initiation may be necessary in certain situations, careful consideration should be given to ensure appropriate and evidence-based prescribing practices.

When measuring the differences in the score of practice in relation to the socio-demographic characteristics of the physicians (Table 4), it was found that a higher practice score was more associated with being older (Z = 3.004; *p* = 0.003), male gender (Z = 2.124; *p* = 0.034), and Saudi nationality (Z = 2.279; *p* = 0.023).

When measuring the differences in the score of practice in regard to the prescribing of antibiotics (Table 5), it was revealed that a higher practice score was more associated with having five cases or fewer of infection cases per day (Z = 2.530; *p* = 0.011) and five or fewer infection cases with initiated antibiotic prescribing treatment (Z = 2.288; *p* = 0.022).

## 4. Discussion

Antimicrobial resistance has indeed emerged as a significant global concern, with increasing prevalence posing a threat to public health worldwide. In response to this growing issue, health authorities have recognized the urgent need for interventions aimed at reducing the prevalence of antimicrobial resistance [15]. One intervention that has gained significant attention and is considered to play a vital role in the healthcare system is the implementation of Antibiotic Stewardship Programs (ASPs). ASPs are comprehensive strategies designed to optimize the use of antibiotics, promoting their judicious and appropriate utilization. ASPs are multifaceted initiatives that involve various interventions and activities. They aim to improve antibiotic prescribing practices, enhance infection control measures, increase awareness and education among healthcare professionals and patients, and implement surveillance systems to monitor and track antimicrobial resistance patterns [16,17].

The current study is one of the first studies conducted in Southern Saudi Arabia that evaluates physicians’ participation in implementing ASPs. The results showed, based on a 37-item questionnaire, that 67.2% of participants were considered to have a good level of ASP practice, 28.1% were moderate, and only 4.7% were considered to have a poor practice level. These findings are at odds with the findings of a previous study conducted by Atif et al., 2021, in Pakistan [3], which reported that the doctors’ perception of ASP was poor, and the existence of activities related to ASP was very limited. Another study by Setiawan et al., 2022, in Indonesia [9], reported that 64.1% of the healthcare professionals were unfamiliar with the term “ASP” and were not keen to participate in any ASP activities. Similarly, another study by Baraka et al., 2019, in the Eastern province of Saudi Arabia [12] revealed that more than half of the clinicians were considered to have unsatisfactory awareness of antimicrobial stewards (AMS) and their elements, with more than seventy percent having no previous experience with AMS. These findings suggest that enhancing knowledge about ASPs among healthcare professionals is crucial for the successful implementation and practice of ASPs. Comprehensive education and training programs can play a vital role in improving understanding and promoting best practices related to ASPs. In Indonesia, pharmacists were more likely to attend and commit to educational activities related to ASP compared to physicians (*p* < 0.05) [9]. In addition to education and training sessions, other activities can be implemented to enhance knowledge and practice of ASPs. These may include workshops, seminars, webinars, and conferences focused on ASPs and antimicrobial stewardship. These platforms provide opportunities for healthcare professionals to share experiences, learn from experts in the field, and discuss challenges and solutions related to ASP implementation [18]. Furthermore, incorporating ASP education and training into the curriculum of medical and healthcare-related educational programs can help instill the principles of antimicrobial stewardship from the early stages of professional development. This can ensure that future healthcare professionals are adequately equipped with the knowledge and skills to practice ASPs effectively [19].

Data in the present study suggest that older age groups, gender male, Saudi nationality, handling five or less than five infection cases daily, or initiating antibiotic prescribing treatment daily were identified as the significant predictors of increased ASP practice. In Canada, a similar study was conducted among antimicrobial prescribers including physicians, residents, and fellows in three teaching hospitals within the Université de Montréal network. The study revealed that the residents tended to choose a broader range of antibiotics (*p* < 0.001) [18]. Approximately 25% of participants were unaware of the presence of an ASP in their respective hospitals. In total, 27% of participants lacked knowledge regarding the location of resources inside their own hospital that would aid in the improvement of antibiotic prescription. However, the differences in the ASP knowledge between the antibiotic prescribers were not statistically significant.

In the specific assessment of physicians’ practice toward the effectiveness of ASP, our results indicate that out of 5 points, the rating was highest in the controlling source of infection domain, followed by enhancing infection domain, and prescribing antibiotics when they are truly needed domain. Other domains were also satisfactory in ratings, including prescribing appropriate antibiotics with adequate dosages, using the shortest duration of antibiotics based on evidence, reassessing antimicrobial treatment when culture results are available, supporting an interdisciplinary approach, educating staff, and supporting surveillance of AMR and HAIs and monitoring of antibiotic consumption.

In the educating staff domain, the mean score for receiving ASP training/orientation was 3.54, while the mean score for receiving feedback to improve prescribing behavior was 3.66, showing a moderate degree of agreement. However, nearly a quarter of participants stated that they “never” or “rarely” received ASP training/orientation (24.2%), as well as feedback to improve prescribing behavior (15.6%). Similarly, the mean score for having received training that provided the necessary confidence, skills, and expertise in antibiotic management was 3.81. While the mean score indicates a moderate level of agreement, a significant percentage of respondents disagreed (13.3%). This implies that certain barriers are impeding the effective practice of ASP, which should be addressed through interventions to improve its effectiveness.

Regarding the surveillance and monitoring of AMR and HAIs domain, the mean scores for supporting the surveillance of antimicrobial resistance (AMR) control, healthcare-associated infections (HAIs), and reporting any identified AMR and HAIs to the antibiotic stewardship program were 3.48, 3.47, and 3.56, respectively. The results suggest a moderate degree of agreement, although a significant proportion of respondents (about 30%) expressed disagreement with supporting surveillance of AMR and HAIs, as well as monitoring antibiotic consumption, in comparison to those who agreed or strongly agreed.

Furthermore, in the interdisciplinary approach domain, the mean score for supporting an interdisciplinary approach was relatively higher at 3.88, indicating a higher level of agreement compared to other domains. The results indicate that the level of support provided by healthcare institution administration for ASPs and infection control was moderate, with mean scores of 3.96 and 4.27, respectively. Similarly, the involvement of pharmacists and staff nurses in ASP was also rated as moderate, with mean scores of 4.02 and 3.58, respectively. This suggests a moderate level of agreement, but a notable number of respondents expressed disagreement with the involvement of these healthcare professionals. In terms of timely reporting of microbiology results and provision of surveillance data on Antimicrobial Resistance (AMR), the mean scores were moderate, with values of 3.71 and 3.72, respectively. While there was a moderate level of agreement among respondents, a considerable number of individuals disagreed with the support provided. Around 12.5% of participants said they “never” or “rarely” received timely and accurate reporting of microbiology susceptibility test results. Similarly, 18% of respondents said they “never” or “rarely” received surveillance data on antimicrobial resistance periodically. These findings highlight some degree of impediment/barriers to the rational prescribing of antibiotics. Hence, there is a need for further improvement and support in various aspects of healthcare institution administration, pharmacist and nurse involvement, as well as timely reporting and surveillance data in relation to ASP and infection control.

Every practice domain mean score was greater than 3, indicating that study participants possessed a moderate level of ASP practice and implementation skills. Overall, the assessment indicates that there is room for improvement in physicians’ practice in the efficacy of ASP. In a study conducted by Haseeb et al., 2020, in the Makkah region [1], the use of clinical guidelines and pathways (100%), formulary restrictions (90%), use of broad-spectrum antimicrobials and prospective feedback on prescribing antimicrobials (68%), and use of automatic stop orders to limit inappropriate antimicrobial therapy (68%) were the most commonly reported ASP practices in Makkah hospitals. They identified education and training as key elements of successful ASPs in Saudi Arabian hospitals. The most common methods for educating doctors, pharmacists, and nurses included workshops, lectures, posters, lunchtime talks, and in-person interventions. They also identified that multidisciplinary antimicrobial stewardship committees, infection control, and surveillance-related activities were vital for successful antimicrobial stewardship programs in Makkah region hospitals. These findings corroborate the findings of the present study.

Barriers and impediments can have a significant impact on the implementation and effectiveness of Antimicrobial Stewardship Programs (ASPs). They can hinder the adoption of appropriate antimicrobial prescribing practices, limit the success of stewardship interventions, and contribute to the persistence of antimicrobial resistance. Numerous studies have addressed the obstacles and challenges encountered during the implementation of ASPs. These research endeavors have focused on identifying and examining the barriers and concerns that arise when implementing ASPs [20,21,22,23,24]. For instance, Howard et al., 2015 [6], reported that lack of personnel, funding, information technology, and prescriber opposition were the major barriers to implementing AMS. Salem et al., 2023, in Egypt [7] documented that the main challenges of ASP include lack of time in implementation, monitoring, and lack of knowledge of the need for ASP. Another study by Alghamdi et al., 2019, in Saudi hospitals [13] reported significant barriers to ASP implementation including the lack of policies and guidelines enforcement, disintegration of teams, lack of ASP team members, poor communication, lack of health information technology, and lack of education and training. In the current study, the educating staff domain also obtained the lowest score, but it was still at a satisfactory level (mean score: 3.67 out of 5 points) and was not posed as a significant challenge among the study participants.

In the current study, regarding the assessment of physician’s practice regarding antibiotic prescribing, it was found that the average number of infection cases managed daily by the physicians was 13.5; the average number of infection cases managed daily with symptomatic treatment without initiating antibiotics was 5.98; the average number of infection cases manage daily with delayed antibiotic prescribing treatment was 3.58; and the average number of infection cases managed daily with initiated antibiotic prescribing treatment was 6.43. These findings indicate that physicians, on average, manage a moderate number of infection cases daily. This suggests that they are regularly encountering patients with infectious conditions. Interestingly, a significant proportion of infection cases are managed with symptomatic treatment alone, without the use of antibiotics. This conservative approach aligns with the principles of Antibiotic Stewardship Programs, aiming to reduce unnecessary antibiotic use and mitigate the development of antibiotic resistance. It indicates that physicians are making efforts to manage infections through non-antibiotic measures, such as supportive care and symptom relief. Moreover, the results demonstrate that delayed antibiotic prescribing is a common strategy employed by physicians. This approach involves providing patients with a prescription but advising them to delay antibiotic use unless their symptoms worsen or fail to improve within a specified timeframe. The high usage of delayed antibiotic prescribing indicates a positive trend towards judicious antibiotic use, as it helps balance the need for prompt treatment with the goal of minimizing unnecessary antibiotic exposure.

However, the findings also reveal that a substantial number of infection cases are treated with initiated antibiotic prescribing. While initiating antibiotics in these cases might be necessary, as in the case of empirical therapy, it highlights the need for continued education and interventions to optimize evidence-based antibiotic prescribing practices. It is essential to ensure that antibiotics are prescribed based on clinical indications, taking into account the potential risks of antibiotic resistance and adverse effects associated with their use. The results indicate a combination of conservative and proactive antibiotic prescribing practices among physicians. The prevalence of symptomatic treatment and delayed antibiotic prescribing reflects a positive trend toward antibiotic stewardship. However, the significant use of initiated antibiotic prescribing suggests room for improvement in optimizing antibiotic use and promoting evidence-based prescribing practices.

A study conducted by Ibrahim and Bazzi, 2021 [19], reported that over eighty percent of clinicians thought that the ASP program was improving antibiotic use, leading to improving the overall quality of care of hospitalized patients. Another study conducted by Kim et al., 2019 [23], in South Korea, reported that Korean hospitals widely implemented restrictive measures for designated antimicrobials, with 88.1% of hospitals adopting such measures. However, the proportion of hospitals implementing interventions to address inappropriate long-term antimicrobial use and strategies for converting from parenteral to oral antimicrobial administration was significantly lower, at only 9.5% and 1.2%, respectively. The major barriers perceived in establishing an ASP in Korean hospitals were identified as a lack of time, personnel, and appropriate compensation.

Potential interventions and strategies to improve physicians’ practice in the efficacy of ASPs:

To improve physicians’ practice in the efficacy of the Antimicrobial Stewardship Program (ASP), several potential interventions and strategies can be implemented as follows: (i) Education and Training: Provide comprehensive education and training programs for physicians on antimicrobial stewardship principles, guidelines, and best practices. (ii) Clinical Decision Support Systems: Implement clinical decision support systems (CDSS) within electronic health records to provide real-time guidance to physicians regarding appropriate antimicrobial prescribing. (iii) Feedback and Audit: Establish systems to provide regular feedback and audit data to physicians about their antimicrobial prescribing practices. This feedback can include information on their individual prescribing patterns, adherence to guidelines, and outcomes related to infection management. (iv) Antibiotic Guidelines and Pathways: Develop and disseminate clear, evidence-based antibiotic guidelines and clinical pathways that outline optimal antimicrobial prescribing practices for different infectious diseases. (v) Antimicrobial Stewardship Team Collaboration: Foster collaboration and communication between physicians and the multidisciplinary antimicrobial stewardship team, including infectious disease specialists, clinical pharmacists, microbiologists, and infection preventionists. This collaboration can facilitate shared decision-making, exchange of knowledge, and integration of stewardship principles into clinical practice. (vi) Champion Leadership: Identify physician champions who have a strong interest and expertise in antimicrobial stewardship. These champions can serve as role models, advocates, and educators within the healthcare facility, encouraging their peers to embrace stewardship practices and demonstrating the positive impact of such initiatives. (vii) Continuing Medical Education: Incorporate antimicrobial stewardship topics into continuing medical education (CME) programs and professional development activities for physicians. This can help reinforce knowledge, raise awareness about emerging resistance patterns, and promote the implementation of stewardship practices. (viii) Performance Incentives: Consider implementing performance incentives or recognition programs that reward physicians who demonstrate adherence to antimicrobial stewardship practices. Recognition can be in the form of financial incentives, public acknowledgement, or career advancement opportunities.

## 5. Conclusions

In conclusion, this study assessed the practice of physicians in Najran city, Saudi Arabia, regarding the Antibiotic Stewardship Program and appropriate antibiotic prescribing. While controlling the source of infection domain received the highest score, supporting surveillance of antimicrobial resistance, healthcare-associated infections, and monitoring antibiotic consumption scored the lowest. The overall practice score was moderate, with a majority of physicians demonstrating good practice. Factors such as older age, male gender, Saudi nationality, handling a lower number of infection cases daily, and infection-initiated antibiotic prescribing treatment were associated with higher practice scores. The findings indicate that there is room for improvement in the implementation of ASP practices among physicians in the southern region of Saudi Arabia. In addition, these findings suggest the need for targeted interventions and educational programs to enhance physicians’ adherence to ASP guidelines and promote appropriate antibiotic prescribing practices, ultimately contributing to the global efforts in combating antimicrobial resistance and improving patient outcomes. It is important to note that a comprehensive and multi-faceted approach is typically most effective in improving physicians’ practice in ASP. The customization of interventions based on the local context, regular evaluation of outcomes, and ongoing feedback and support are crucial for sustaining improvements in ASP practices.

Supplementary file: The study toll questionnaire used for data collection.

## Figures and Tables

**Table 1:** Socio-demographic characteristics of the physicians (*n* = 128).

Study Variables	*n* (%)
Age group	
24–35 years	50 (39.1%)
36–45 years	56 (43.8%)
>45 years	22 (17.2%)
Gender	
Male	77 (60.2%)
Female	51 (39.8%)
Marital status	
Single	35 (27.3%)
Married	93 (72.7%)
Nationality ^†^	
Saudi	38 (31.1%)
Non-Saudi	84 (68.9%)
Position ^†^	
Supervisor	13 (12%)
Practitioner	75 (69.4%)
Both	20 (18.5%)
Qualifications	
Diploma	43 (33.6%)
Bachelor	5 (3.9%)
Master	21 (16.4%)
PhD	8 (6.3%)
Board	42 (32.8%)
Others	9 (7%)
Healthcare facility ^†^	
PHC Center	16 (13.1%)
Hospital-Outpatient Clinic	54 (44.3%)
Hospital-Emergency Department	47 (38.5%)
Polyclinic	5 (4.1%)
Workplace ^†^	
MOH	67 (54%)
University Health Services	16 (12.9%)
Military Health Services	17 (13.7%)
National Guard Health Services	5 (4%)
Interior Ministry Health Services	1 (0.8%)
Private Sector	18 (14.5%)
Years of experience	
1–5 years	40 (31.3%)
6–10 years	32 (25%)
11–15 years	31 (24.2%)
>15 years	25 (19.5%)

^†^ Missing cases were excluded from the analysis.

**Table 2:** Assessment of physicians’ practice in the effective implementation of ASP (*n* = 128).

Statement	Mean ± SD	Never *n* (%)	Rarely *n* (%)	Sometimes *n* (%)	Often *n* (%)	Always *n* (%)
*Enhancing infection prevention and control score*	4.75 ± 0.51	**--**	**--**	**--**	**--**	**--**
I prevent healthcare-associated infections from occurring in the first place	4.75 ± 0.61	1 (0.80%)	0	6 (4.7%)	16 (12.5%)	105 (82.0%)
2. I prevent transmission of healthcare-associated infections when they occur	4.74 ± 0.55	0	0	7 (5.5%)	19 (14.8%)	102 (79.7%)
*Controlling source control score*	4.83 ± 0.39	**--**	**--**	**--**	**--**	**--**
3. I look for the septic source as early as possible	4.86 ± 0.39	0	0	2 (1.6%)	14 (10.9%)	112 (87.5%)
4. I control the verified source of infection as soon as possible	4.8 ± 0.49	0	1 (0.80%)	2 (1.6%)	18 (14.1%)	107 (83.6%)
*Prescribing antibiotics when they are truly needed score*	4.45 ± 0.59	**--**	**--**	**--**	**--**	**--**
5. I use antibiotics after a treatable infection has been recognized.	4.5 ± 0.95	2 (1.6%)	7 (5.5%)	4 (3.1%)	26 (20.3%)	89 (69.5%)
6. I use antibiotics when there is a high degree of suspicion of infection.	4.59 ± 0.66	0	1 (0.80%)	9 (7.0%)	32 (25.0%)	86 (67.2%)
7. I provide advice on prudent antibiotic use to individuals.	4.37 ± 0.95	3 (2.3%)	0	17 (13.3%)	33 (25.8%)	75 (58.6%)
8. I use good negation skills to convince individuals about unnecessary antibiotic use	4.36 ± 0.99	4 (3.1%)	6 (4.7%)	6 (4.7%)	36 (28.1%)	76 (59.4%)
*Prescribing appropriate antibiotics with adequate dosages score*	4.43 ± 0.79	**--**	**--**	**--**	**--**	**--**
9. I Initiate empirical antibiotic therapy in patients who need immediate treatment.	4.52 ± 1.05	5 (3.9%)	0	8 (6.3%)	22 (17.2%)	93 (72.7%)
10. I Initiate empirical antibiotic therapy based on local epidemiology.	4.16 ± 1.17	4 (3.1%)	8 (6.3%)	18 (14.1%)	28 (21.9%)	70 (54.7%)
11. I Initiate empirical antimicrobial therapy based on individual patient risk factors for difficult-to-treat pathogens and clinical severity of infection.	4.45 ± 0.91	2 (1.6%)	3 (2.3%)	8 (6.3%)	35 (27.3%)	80 (62.5%)
12. I Initiate empirical antibiotic therapy based on the infection source.	4.42 ± 1.05	4 (3.1%)	3 (2.3%)	10 (7.8%)	26 (20.3%)	85 (66.4%)
13. I take into account the antibiotic resistance rates when initiating empirical antimicrobial therapy	4.39 ± 0.98	3 (2.3%)	1 (0.80%)	13 (10.2%)	34 (6.6%)	77 (60.2%)
14. I take in account the previous antibiotic when initiating empirical antimicrobial therapy	4.57 ± 0.94	3 (2.3%)	0	10 (7.8%)	20 (7.8%)	95 (74.2%)
15. I establish antibiotic dosing regimens based on host factors and properties of antibiotic	4.51 ± 0.99	4 (3.1%)	0	6 (4.7%)	31 (24.2%)	87 (68.0%)
*Reassessing antimicrobial treatment when culture results are available score*	4.33 ± 0.87	**--**	**--**	**--**	**--**	**--**
16. I reassess antibiotic therapy when the culture and susceptibility results are available	4.69 ± 0.78	2 (1.6%)	0	5 (3.9%)	20 (15.6%)	101 (78.9%)
17. I would expand antibiotic therapy if the empirical choice were too narrow	4.16 ± 1.08	3 (2.3%)	5 (3.9%)	21 (16.4%)	35 (27.3%)	64 (50.0%)
18. I de-escalate antibiotic therapy if the empirical regimen was too broad	4.13 ± 1.24	6 (4.7%)	5 (3.9%)	15 (11.7%)	36 (28.1%)	66 (51.6%)
*Using the shortest duration of antibiotics based on evidence score*	4.42 ± 0.85	**--**	**--**	**--**	**--**	**--**
19. I establish antibiotic short duration as much as possible unless there are special circumstances, e.g., immunosuppression or ongoing infections.	4.49 ± 0.88	2 (1.6%)	1 (0.80%)	10 (7.8%)	32 (25.0%)	83 (64.8%)
20. I use oral antibiotics as substitute IV agents as soon as the patient is tolerating an oral diet.	4.40 ± 0.99	3 (2.3%)	2 (1.6%)	15 (11.7%)	27 (21.1%)	81 (63.3%)
21. When conversion to an oral antibiotic, I consider antibiotics having high oral bioavailability (e.g., fluoroquinolones)	4.23 ± 0.99	2 (1.6%)	6 (4.7%)	13 (10.2%)	44 (34.4%)	63 (49.2%)
22. For patients who have signs of sepsis beyond 5 days of treatment, I consider warranting aggressive diagnostic investigation to determine if an ongoing uncontrolled source of infection or antibiotic treatment failure is present	4.55 ± 1.11	6 (4.7%)	0	3 (2.3%)	22 (17.2%)	97 (75.8%)
*Educating staff score*	3.67 ± 1.27	**--**	**--**	**--**	**--**	**--**
23. I get training/orientation in antimicrobial stewardship program (ASP)	3.54 ± 1.48	16 (12.5%)	15 (11.7%)	25 (19.5%)	24 (18.8%)	48 (37.5%)
24. I get feedback to improve my prescribing behavior continuously	3.66 ± 1.39	10 (7.8%)	10 (7.8%)	35 (19.5%)	25 (19.5%)	48 (37.5%)
25. I have training that equips the required confidence, skills, and expertise in the field of antibiotic management	3.81 ± 1.35	9 (7.0%)	8 (6.3%)	28 (21.9%)	30 (23.4%)	53 (41.4%)
*Supporting surveillance of AMR and HAIs and monitoring of antibiotic consumption score*	3.56 ± 1.34	**--**	**--**	**--**	**--**	**--**
26. I have a key role in supporting the antibiotic resistance control	3.74 ± 1.38	13 (10.2%)	7 (5.5%)	26 (20.3%)	32 (25.0%)	50 (39.1%)
27. I survey antimicrobial resistance (AMR).	3.48 ± 1.49	18 (14.1%)	11 (8.6%)	28 (21.9%)	28 (21.9%)	43 (33.6%)
28. I survey healthcare-associated infections (HAIs).	3.47 ± 1.56	18 (14.1%)	18 (14.1%)	16 (12.5%)	31 (24.2%)	45 (35.2%)
29. If I detect AMR and HAIs, I notify the antimicrobial stewardship program (ASP) team members	3.56 ± 1.52	20 (15.6%)	10 (7.8%)	21 (16.4%)	28 (21.9%)	49 (38.3%)
*Supporting an interdisciplinary approach score*	3.88 ± 1.09	**--**	**--**	**--**	**--**	**--**
30. There is collaboration between all healthcare professionals to share knowledge and practice to succeed ASP	3.95 ± 1.28	8 (6.3%)	6 (4.7%)	22 (17.2%)	35 (27.3%)	57 (44.5%)
31. Healthcare institution administration provides adequate support for both developing and sustaining an ASP	3.96 ± 1.27	7 (5.5%)	7 (5.5%)	22 (17.2%)	35 (27.3%)	57 (44.5%)
32. Infection control department/unit monitor and prevent healthcare-associated infections	4.27 ± 1.02	3 (2.3%)	3 (2.3%)	15 (11.7%)	40 (31.3%)	67 (52.3%)
33. Pharmacists are key actors in the design and implementation of the stewardship program	4.02 ± 1.33	11 (8.6%)	2 (1.6%)	16 (12.5%)	38 (29.7%)	61 (47.7%)
34. Pharmacists provide feedback to physicians about prudent antibiotic use	3.83 ± 1.32	10 (7.8%)	7 (5.5%)	22 (17.2%)	40 (31.3%)	49 (38.3%)
35. The staff nurses integrate antimicrobial stewardship	3.58 ± 1.43	17 (13.3%)	4 (3.1%)	30 (23.4%)	36 (28.1%)	41 (32.0%)
36. Timely and accurate reporting of microbiology susceptibility test results is available	3.71 ± 1.33	12 (9.4%)	4 (3.1%)	36 (28.1%)	29 (22.7%)	47 (36.7%)
37. Surveillance data on antimicrobial resistance are provided periodically	3.72 ± 1.38	11 (8.6%)	12 (9.4%)	26 (20.3%)	28 (21.9%)	51 (39.8%)
Total practice score	154.9 ± 25.5	**--**	**--**	**--**	**--**	**--**
Level of practice in the efficacy of ASP						
• Poor	06 (04.7%)	**--**	**--**	**--**	**--**	**--**
• Moderate	36 (28.1%)	**--**	**--**	**--**	**--**	**--**
• Good	86 (67.2%)	**--**	**--**	**--**	**--**	**--**

Response has a range from “Never” coded with 1 to “Always” coded with 5.

**Table 3:** Assessment of physicians’ practice regarding the prescribing of antibiotics (*n* = 128).

Variables	*n* (%)
Average number of infection cases you manage daily (mean ± SD)	13.5 ± 14.1
≤5	52 (40.6%)
6–10	17 (13.3%)
11–20	36 (28.1%)
>20	23 (18.0%)
Average number of infection cases you manage daily with symptomatic treatment (no antibiotic) (mean ± SD)	5.98 ± 8.11
≤5	82 (64.1%)
6–10	28 (21.9%)
11–20	16 (12.5%)
>20	2 (1.6%)
Average number of infection cases you manage daily with delayed antibiotic prescribing treatment (mean ± SD)	3.58 ± 4.29
≤5	103 (80.5%)
6–10	23 (18.0%)
11–20	2 (1.6%)
>20	0
Average number of infection cases you manage daily with initiated antibiotic prescribing treatment (mean ± SD)	6.43 ± 6.65
≤5	80 (62.5%)
6–10	26 (20.3%)
11–20	18 (14.1%)
>20	4 (3.1%)

**Table 4:** Differences in practice score and the socio-demographic characteristics of the physicians (*n* = 128).

Factor	Practice Score (185) Mean ± SD	Z-Test	*p*-Value ^§^
Age group			
<40 years	150 ± 28.6	3.004	0.003 **
≥40 years	164 ± 14.9		
Gender			
Male	159.8 ± 18.9	2.124	0.034 **
Female	147.9 ± 31.8		
Marital status			
Single	157.9 ± 20.2	0.3	0.764
Married	153.8 ± 27.3		
Nationality ^†^			
Saudi	163 ± 15.9	2.279	0.023 **
Non-Saudi	150.9 ± 28.3		
Position ^†^			
Supervisor	157.8 ± 19.2	3.34	0.188 ^‡^
Practitioner	150.5 ± 28.6		
Both	162.1 ± 6.4		
Qualifications			
Bachelor or diploma	156.9 ± 25.9	0.81	0.418
Master or higher	153.7 ± 25.3		
Healthcare facility ^†^			
PHC Center/Polyclinic	161.9 ± 17.7	0.657	0.72
Hospital-Outpatient Clinic	152.5 ± 30.3		
Hospital-Emergency Department	153.9 ± 23.7		
Workplace ^†^			
MOH	151.9 ± 26.7	0.903	0.366
Non-MOH	157.8 ± 19.9		
Years of experience			
≤10 years	156.6 ± 24.4	0.606	0.545
>10 years	152.8 ± 26.9		

^†^ Not mentioned cases were excluded from the analysis. ^§^ *p*-value has been calculated using Mann–Whitney Z-test. ^‡^ *p*-value has been calculated using Kruskal–Wallis H-test. ** Significant at *p* < 0.05 level.

**Table 5:** Differences in practice score and the practice of the physicians regarding prescribing antibiotics (*n* = 128).

Factor	Practice Score (185) Mean ± SD	Z-Test	*p*-Value ^§^
Average number of infections cases you manage daily			
≤5	161.8 ± 19.4	2.53	0.011 **
>5	150.2 ± 28.2		
Average number of infection cases you manage daily with symptomatic treatment (no antibiotic)			
≤5	155.5 ± 25.4	0.318	0.75
>5	153.9 ± 25.9		
Average number of infection cases you manage daily with delayed antibiotic prescribing treatment			
≤5	154.5 ± 24.2	1.23	0.219
>5	156.8 ± 30.9		
Average number of infection cases you manage daily with initiated antibiotic prescribing treatment			
≤5	158.4 ± 23.9	2.288	0.022 **
>5	149.2 ± 27.3		

^§^ *p*-value has been calculated using Mann–Whitney Z-test. ** Significant at *p* < 0.05 level.

## Data Availability

The data that support the findings of this study are available with the corresponding author upon reasonable request.

## Supplementary Materials

The following supporting information can be downloaded at: https://www.mdpi.com/article/10.3390/pharmacy12010024/s1.
