# Supplementary material for: Assessment of Physicians’ Practice in Implementing Antibiotic Stewardship Program in Najran City, Saudi Arabia: A Cross-Sectional Study
Source: Pharmacy (Basel). 2024 Feb 1;12(1):24. doi: 10.3390/pharmacy12010024 (PMC10892749; doi:10.3390/pharmacy12010024)
Supplement: Supplementary file 1 [file pharmacy-12-00024-s001.zip › pharmacy-2745377-supplementary.pdf]

DATA COLLECTION QUESTIONNAIRE

FIRST: SOCIO-DEMOGRAPHIC CHARACTERISTICS:

1. Age: ………. years
2. Gender: ▢ Male ▢ Female
3. Family status: ▢ Single ▢ Married
4. Nationality: ▢ Saudi ▢ Non-Saudi
5. Position: ▢ Supervisor ▢ Practitioner ▢ Both
6. Qualifications: ▢ Bachelor ▢ Diploma ▢ Master
   ▢ PhD ▢ Board ▢ Other (specify…………….)
7. Healthcare facility: ▢ PHC center ▢ Hospital-outpatient clinic
   ▢ Hospital-emergency department ▢ Polyclinic
8. Work place: ▢ MOH ▢ University health services
   ▢ Military health services ▢ National guard health services
   ▢ Interior ministry health services ▢ Private sector
9. Years of experience (…………)

SECOND PART: PHYSICIAN'S PARTICIPATION IN EFFICACY OF ASP

	Practice of physician	Response				
		Always	Often	Sometimes	Rarely	Never
**1st: Enhancing infection prevention and control**						
1	I prevent healthcare associated infections from occurring in the first place.					
2	I prevent transmission of healthcare associated infections when occur.					
**2nd: Controlling source control**						
1	I look for the septic source as early as possible.					
2	I control the verified source of infection as soon as possible.					
**3rd: Prescribing antibiotics when they are truly needed**						
1	I use the antibiotics after a treatable infection has been recognized.					
2	I use the antibiotics when there is a high degree of suspicion for infection.					
3	I provide advice on prudent antibiotic use to individuals.					
4	I have good negation skills to convince individuals about unnecessary antibiotic use.					
**4th: Prescribing appropriate antibiotics with adequate dosages**						
1	I Initiate empirical antibiotic therapy in patients need immediate treatment.					
2	I Initiate empirical antibiotic therapy based on local epidemiology.					
3	I Initiate empirical antimicrobial therapy based on individual patient risk factors for difficult to treat pathogens and clinical severity of infection.					
4	I Initiate empirical antibiotic therapy based on the infection source.					
5	I take in account the antibiotic resistance rates when initiate empirical antimicrobial therapy.					
6	I take in account the previous antibiotic when initiate empirical antimicrobial therapy.					

7	I establish antibiotic dosing regimen based on host factors and properties of antibiotic.					
**5th: Reassessing antimicrobial treatment when culture results are available**						
1	I reassess antibiotic therapy when the culture and susceptibility results are available.					
2	I expand antibiotic therapy if the empirical choice was too narrow.					
3	I de-escalation of antibiotic therapy if the empirical regimen was too broad.					
**6th: Using the shortest duration of antibiotics based on evidence**						
1	I establish antibiotic duration short as much as possible unless there are special circumstances e.g. immunosuppression, or ongoing infections.					
2	I use oral antibiotic as substitute IV agents as soon as the patient is tolerating an oral diet.					
3	when conversion to oral antibiotic I consider antibiotic having high oral bioavailability (e.g. fluoroquinolones)					
4	For patients who have signs of sepsis beyond 5 days of treatment I consider warrant aggressive diagnostic investigation to determine if an ongoing uncontrolled source of infection or antibiotic treatment failure is present.					
**7th: Educating staff**						
1	I get training /orientation in antimicrobial stewardship program (ASP).					
2	I get feedback to improve my prescribing behavior continuously.					
3	I have training that equip the required confidence, skills and expertise in the field of antibiotic management.					
**8th: Supporting surveillance of AMR and HAIs and monitoring of antibiotic consumption**						
1	I have a key role in supporting the antibiotic resistance control.					
2	I survey antimicrobial resistance(AMR).					
3	I survey healthcare-associated infections (HAIs).					
4	If I detect AMR and HAIs ; I notify to antimicrobial stewardship program (ASP) team members.					
**9th: Supporting an interdisciplinary approach**						

1	There is collaboration between all healthcare professionals to shared knowledge and practice to succeed ASP.					
2	Healthcare institution administration provides adequate support for both developing and sustaining an ASP.					
3	Infection control department/unit monitor and prevent healthcare-associated infections.					
4	Pharmacists are key actors for the design and implementation of the stewardship program.					
5	Pharmacists provide feedback to physician about prudent antibiotic use.					
6	The staff nurses integrate in antimicrobial stewardship.					
7	Timely and accurate reporting of microbiology susceptibility test results are available					
8	Surveillance data on antimicrobial resistance are provided periodically					

<u>THIRD PART: Practice of physicians regarding a prescribing of antibiotics</u>

	**DAILY PRESCRIBING ANTIBIOTICS**	**NUMBER**
1	Average number of infection cases you manage daily.	
2	Average number of infection cases you manage daily with symptomatic treatment (NO ANTIBIOTIC).	
3	Average number of infection cases you manage daily with delayed antibiotic prescribing treatment.	
4	Average number of infection cases you manage daily with initiated antibiotic prescribing treatment.	
